# Early and late CTCAE and patient-reported outcomes following lung cancer radiotherapy: A sex-stratified descriptive analysis from the REQUITE cohort

**DOI:** 10.1016/j.ctro.2026.101265

**Published:** 2026-08-20

**Authors:** Miguel E. Aguado-Barrera, Patricia Calvo-Crespo, Begoña Taboada-Valladares, Antonio Gomez-Caamaño, Angel Carracedo, David Azria, Erik Briers, Jenny Chang-Claude, Dirk de Ruysscher, Gilles Defraene, Rebecca M. Elliott, Corinne Faivre-Finn, Marzia Franceschini, Olivia Fuentes-Rios, Alexandra Giraldo, Tommaso Giandini, Sara Gutiérrez-Enríquez, Philipp Heumann, Daniel S. Higginson, Kerstie Johnson, Maarten Lambrecht, Yolande Lievens, Meritxell Mollà, Tiziana Rancati, Tim Rattay, Mónica Ramos, Barry S. Rosenstein, Petra Seibold, Elena Sperk, Hilary Stobart, Paul Symonds, Christopher J. Talbot, Katrien Vandecasteele, Liv Veldeman, Tim Ward, Adam Webb, Catharine M.L. West, REQUITE consortium, Vega Ana

**Affiliations:** a Grupo Genética en Cáncer y Enfermedades Raras, Instituto de Investigación Sanitaria de Santiago de Compostela, Santiago de Compostela, Spain; b Fundación Pública Galega de Medicina Xenómica, Santiago de Compostela, Spain; c Department of Radiation Oncology, Hospital Clínico Universitario de Santiago de Compostela, Servizo Galego de Saúde (SERGAS), Santiago de Compostela, Spain; d Genomic Medicine Group, CIMUS, University of Santiago de Compostela, Santiago de Compostela, Spain; e Biomedical Network on Rare Diseases (CIBERER), Instituto de Salud Carlos III, Madrid, Spain; f Genetics Group, Instituto de Investigación Sanitaria de Santiago de Compostela, Santiago de Compostela, Spain; g Fédération Universitaire d'oncologie radiothérapie d'Occitanie Méditerranée, ICM, INSERM U1194 IRCM, Univ Montpellier, Montpellier, France; h Patient Advocat, Hasselt, Belgium; i Division of Cancer Epidemiology, German Cancer Research Center (DKFZ), Heidelberg, Germany; j Cancer Epidemiology Group, University Cancer Center Hamburg, University Medical Center Hamburg-Eppendorf, Hamburg, Germany; k Maastricht University Medical Center, Department of Radiation Oncology (Maastro clinic), GROW School for Oncology and Developmental Biology, Maastricht, the Netherlands; l Department of Oncology, KU Leuven, Leuven, Belgium; m Translational Radiobiology Group, Division of Cancer Sciences, The University of Manchester, Manchester Academic Health Science Centre, Christie Hospital, Manchester, United Kingdom; n Clinical Oncology Department, The Christie NHS Foundation Trust, Manchester, United Kingdom; o Division of Cancer Sciences, The University of Manchester, Manchester, United Kingdom; p Radiation Oncology Unit, Fondazione IRCCS Istituto Nazionale dei Tumori, Milan, Italy; q Department of Radiation Oncology, Vall d'Hebron Hospital Universitari, Vall d'Hebron Barcelona Hospital Campus, Barcelona, Spain; r Medical Physics Unit, Fondazione IRCCS Istituto Nazionale dei Tumori, Milan, Italy; s Hereditary Cancer Genetics Group, Vall d'Hebron Institute of Oncology (VHIO), Vall d'Hebron Barcelona Hospital Campus, Barcelona, Spain; t Department of Radiation Oncology, Memorial Sloan Kettering Cancer Center, New York, NY, USA; u Leicester Cancer Research Centre, University of Leicester, Leicester, United Kingdom; v Department of Radiation Oncology, UZ Gasthuisberg Leuven, Leuven, Belgium; w Radiation Oncology, Ghent University Hospital and Ghent University, Ghent, Belgium; x Unit of Data Science, Fondazione IRCCS Istituto Nazionale dei Tumori, Milan, Italy; y Department of Radiation Oncology, Department of Genetics and Genomic Sciences, Icahn School of Medicine at Mount Sinai, New York, USA; z Department of Radiation Oncology, Universitätsmedizin Mannheim, Medical Faculty Mannheim, University of Heidelberg, Mannheim, Germany; aa Independent Cancer Patients' Voice, United Kingdom; ab Division of Genetics and Genome Biology, University of Leicester, Leicester, United Kingdom; ac Trustee Pelvic Radiation Disease Association, NCRI CTRad Consumer, United Kingdom

**Keywords:** Radiotherapy, adverse events, Patient-reported outcomes, Prospective cohort study, lung cancer, sex-stratified analysis, REQUITE

## Abstract

**Background:**

Long-term prospective data on lung cancer patients treated with radiotherapy are limited, restricting understanding of outcomes and sex-specific characteristics. The multicentre REQUITE study provides standardized follow-up data from an international cohort.

**Methods:**

We analysed longitudinal data from 530 lung cancer patients treated with radical radiotherapy (sequential or concurrent chemoradiotherapy, or stereotactic body radiation therapy [SBRT]) between 2014 and 2017 at 16 centres in Europe and the USA. Healthcare professionals prospectively recorded 21 pulmonary, oesophageal, neurological, cardiac, and skin adverse events using CTCAE v4.0. Patient-reported outcomes assessed symptoms, quality-of-life, fatigue, and physical activity. Adverse event incidence was stratified by sex, radiotherapy technique, chemotherapy administration, smoking status, and other clinical factors.

**Results:**

At 12 months, 309 patients were evaluable, with 151 having follow-up beyond one year. Pulmonary adverse events were most frequent (40% grade ≥ 2), mainly dyspnoea and cough. Women had higher rates of oesophagitis, and more frequently reported dysphagia, and chest wall pain, while men experienced a higher frequency of cardiac adverse events. Exploratory subgroup analyses identified significant sex-related differences in grade ≥ 3 pulmonary adverse events following SBRT and in overall grade ≥ 2 oesophageal adverse events among patients with clinical stage I-II and those aged >70 years. Patient-reported outcomes showed persistent fatigue and reduced physical activity, particularly in females. Symptom prevalence and severity varied by sex, age, treatment modality, smoking status, and clinical stage.

**Conclusion:**

The REQUITE-Lung cohort provides prospectively collected real-world data on radiotherapy-related adverse events in lung cancer patients. This study describes patterns of adverse events and patient-reported outcomes according to sex, age, and treatment characteristics. These findings are hypothesis-generating and may support future validation in independent and pooled datasets.

## Highlights

• REQUITE-Lung data support risk-adapted radiotherapy analyses in lung cancer patients.
• Pulmonary symptoms predominated, with clear sex-based differences in adverse events.
• Women showed higher adverse events and worse subjective physical function after radiotherapy.

## 1 Introduction

Radiotherapy is an established component of lung cancer (LC) management. While surgery remains the standard approach for early-stage disease, radiotherapy represents an effective alternative for patients who are medically inoperable or decline surgery and serves as a primary treatment modality for unresectable disease, often in combination with chemotherapy and/or immunotherapy [1], [2]. The primary side effects associated with LC radiotherapy involve the lungs and oesophagus. Radiation pneumonitis is the main dose-limiting adverse event [3], with the incidence of grade 2 or higher events decreasing from 30 to 35% with three-dimensional conformal radiotherapy to less than 20% with Intensity-Modulated Radiotherapy (IMRT) or Volumetric Modulated Arc Therapy (VMAT) [4], [5]. The key predictors of grade ≥ 2 pneumonitis are dosimetric parameters, particularly the mean lung dose [6], [7] and the percentage of lung volume receiving ≥20 Gy (V20) [8]. Dyspnoea is a challenging endpoint to assess, as nearly 50% of patients experience it prior to undergoing radiotherapy, and it may also be related to underlying comorbidities [9], [10]. Oesophagitis is the most common acute oesophageal adverse event [11], [12], whereas late complications include strictures, ulceration, and tracheoesophageal fistulae [13], [14]. Less common adverse events include cardiac complications, such as pericarditis and myocarditis, and neurological effects, including myelitis and brachial plexopathy [15], [16]. In addition, fatigue is very common during and after radiotherapy, yet data describing its burden in LC patients remain limited despite the availability of validated assessment tools [17], [18], [19], [20].

A major challenge in evaluating radiotherapy outcomes for LC is the lack of prospective, standardized follow-up data. Many radiotherapy-focused LC cohorts are constraints by small sample sizes, leading to substantial selection bias, while meta-analyses are often limited by heterogeneity in study design, data collection processes and phenotype definitions [21]. The REQUITE study [22] addressed this limitation by establishing a a prospective, multicentre database with harmonised data collection to support translational research, including the evaluation and validation of candidate biomarkers and predictive models. Previously, we reported clinical outcomes up to one-year post-radiotherapy [23]. In the present study, we extend these analyses to longer-term follow-up within the REQUITE cohort and describe adverse events and patient-reported outcomes in a real-world setting. Sex-specific patterns in adverse events outcomes were also explored.

The data from this study are available upon request (requite@le.ac.uk) and may be utilized for research purposes. Further details on the prostate and breast cancer cohorts within the REQUITE study are published as part of a dedicated article series in this issue.

## 2 Methods

### 2.1 Study design

The REQUITE-Lung cohort comprised 530 individuals with LC (159 female and 371 male), aged 18 years or older (with no upper age limit), who were enrolled in the international observational REQUITE study [23] between 2014 and 2017. Participants underwent curative-intent radiotherapy, including radical radiotherapy alone, sequential or concurrent chemoradiotherapy, or stereotactic body radiation therapy (SBRT), with or without surgery. Recruitment was conducted in 16 radiation oncology departments in hospital centres across Belgium, France, the UK, Italy, the Netherlands, Spain, and the USA. Exclusion criteria included the presence of metastatic disease, prior lung irradiation, planned use of proton therapy, or treatment with high intensity focal ultrasound. Blood samples were collected prior to treatment for genotyping and biomarkers analysis. The study protocol was approved by the relevant local ethics committees, and written informed consent was obtained from all participants following the provision of detailed study information. Data collection procedures are described in the Supplementary Methods.

### 2.2 Clinical assessments and outcomes

Symptom prevalence was assessed before radiotherapy (pre-RT), at 3 and 6 months after treatment initiation, and annually thereafter for up to six years, with the primary analysis focused on the 12-month follow-up. Extended follow-up beyond two years was available at selected centres for patients who remained alive and available for assessment. Sex was recorded by a healthcare professional during the pre-radiotherapy medical consultation and defined as a set of biological characteristics, including both internal and external physical anatomy. It follows the typical binary classification (male/female) assigned at birth based on the visible external anatomy of the newborn, commonly referred to as “sex assigned at birth.”

Acute adverse events were defined as events occurring from the start of radiotherapy to 6 months after treatment initiation. Late adverse events were defined as events reported between 6 and 12 months, while assessments performed beyond 12 months were grouped as late adverse events occurring more than 12 months after treatment initiation.

The severity of patient-reported symptoms was evaluated relative to baseline, with an event defined as any worsening from the baseline score.

### 2.3 Subgroup analyses

To explore potential differences in radiotherapy outcomes, analyses were performed separately for female and male patients at each assessment.

Cumulative incidence and maximum-grade adverse events rates were stratified by radiotherapy technique (Three-Dimensional Conformal Radiation Therapy (3DCRT), IMRT/ARC/Tomotherapy, SBRT/Stereotactic Ablative Radiotherapy (SABR)), chemotherapy use, smoking status, chronic obstructive pulmonary disease (COPD), clinical stage and age at the 12-month follow-up (median 70 years).

### 2.4 Statistical analysis

This study was designed as a descriptive analysis. Baseline characteristics, adverse events, and patient-reported outcomes were summarized using absolute frequencies and percentages and presented in tables and graphical displays according to follow-up time. Maximum toxicity rates, cumulative incidence, and longitudinal adverse event frequencies were described overall and within predefined subgroups. Comparisons between female and male patients were performed using Pearson's chi-squared test or Fisher's exact test, as appropriate, with *p*-values adjusted for multiple testing using the Benjamini–Hochberg procedure. All statistical analyses and graphical visualizations were performed using R software [24].

## 3 Results

### 3.1 Cohort description and data availability

A total of 530 LC patients were enrolled prior to radiotherapy, 309 completed 12-month follow-up and 151 continued beyond one year (Table 1***,*** ***Suppl. Table S1***). The female-to-male ratio was 0.43 (159/370), with 98% of participants self-identified as White (99% of females, 96% of males). Median age at enrolment was 67 years for females and 70 years for males; among those completing 12-months, median age at radiotherapy was 67 and 71 years, respectively. At treatment initiation, 21% of male participants were current smokers. Among those with available data at the 12-month follow-up, 17% were current smokers. In female participants, the proportion of current smokers was 25% at treatment initiation and remained 25% among those assessed at 12 months. These figures reflect the characteristics of participants with available follow-up data rather than changes in individual smoking status over time. At radiotherapy start, 41% of both sexes had COPD; among those followed beyond 12 months, the proportion with COPD was 50% in females and 47% in males.

**Table 1:** Baseline Characteristics of REQUITE Lung Cancer Patients: Pre-Treatment (Pre-RT), 12 Months, and Beyond 12 Months Post-Radiotherapy (Post-RT).

	**Pre-RT** (*N* = 530)		**12 months Post-RT** (*N* = 310)		**> 12 months Post-RT** (*N* = 151)	
	**female** (*N* = 159) N (%)	**male** (*N* = 371) N (%)	**female** (*N* = 91) N (%)	**male** (*N* = 219) N (%)	**female** (*N* = 48) N (%)	**male** (*N* = 103) N (%)
**Age (yrs)**						
median (IQR)	67.0 (14.0)	70.0 (15.0)	67.0 (17.0)	71.0 (14.0)	63.8 (18.2)	70.5 (15.0)
range	[39–89]	[39–91]	[47–87]	[45–91]	[39–87]	[48–91]
**Country**						
Belgium	36 (22.6)	103 (27.8)	23 (25.3)	74 (33.8)	17 (35.4)	44 (42.7)
France	13 (8.2)	39 (10.5)	9 (9.9)	25 (11.4)	5 (10.4)	16 (15.5)
Italy	12 (7.5)	35 (9.4)	4 (4.4)	20 (9.1)	–	2 (1.9)
Netherlands	24 (15.1)	37 (10)	14 (15.4)	21 (9.6)	8 (16.7)	16 (15.5)
Spain	22 (13.8)	89 (24)	11 (12.1)	41 (18.7)	6 (12.5)	8 (7.8)
UK	36 (22.6)	55 (14.8)	20 (22)	31 (14.2)	9 (18.8)	13 (12.6)
USA	16 (10.1)	13 (3.5)	10 (11)	7 (3.2)	3 (6.3)	4 (3.9)
**Smoker status**						
Never	15 (9.4)	8 (2.2)	9 (9.9)	3 (1.4)	5 (10.4)	–
Former	104 (65.4)	281 (75.7)	60 (65.9)	175 (79.9)	31 (64.6)	84 (81.6)
Current	40 (25.2)	79 (21.3)	22 (24.2)	39 (17.8)	12 (25)	18 (17.5)
Missing	–	3 (0.8)	–	2 (0.9)	–	1 (1)
**Chronic obstructive pulmonary disease**						
Yes	66 (41.5)	154 (41.5)	44 (48.4)	86 (39.3)	24 (50)	49 (47.6)
**Clinical stage at diagnosis**						
I, II	70 (44.03)	161 (43.51)	48 (52.75)	111 (50.68)	27 (56.25)	59 (57.28)
IIIa, IIIb	86 (54.09)	201 (54.32)	43 (47.25)	101 (46.12)	21 (43.75)	41 (39.81)
missing	3 (1.89)	8 (2.16)	0 (0)	7 (3.2)	0 (0)	3 (2.91)
**Chemotherapy**						
yes	88 (55.3)	184 (49.7)	44 (48.4)	99 (45.2)	22 (45.8)	41 (39.8)
**Radiotherapy technique**						
3D CRT	41 (25.8)	120 (32.4)	18 (19.8)	53 (24.2)	7 (14.6)	17 (16.5)
ARC therapy (VMAT, Rapid ARC)	25 (15.7)	45 (12.2)	10 (11.0)	22 (10.0)	1 (2.1)	10 (9.7)
IMRT	47 (29.6)	93 (25.1)	26 (28.6)	54 (24.7)	17 (35.4)	26 (25.2)
Tomotherapy	2 (1.3)	9 (2.4)	1 (1.1)	9 (4.1)	0 (0)	1 (1.0)
Stereotactic body radiotherapy	44 (27.7)	103 (27.8)	36 (39.6)	81 (37.0)	23 (47.9)	49 (47.6)

Abbreviations: Pre-RT = Pre-Radiotherapy; Post-RT = post-Radiotherapy; N = number of observations; IQR = interquartile range; UK=United Kingdom; USA = United States of America; yrs. = years; 3D CRT = Three Dimensional (3D) Conformal Radiation Therapy; VMAT = Volumetric Modulated Arc Therapy; IMRT = Intensity-Modulated Radiotherapy.

As the study population evolve, the distribution of baseline characteristics among participants varied across time points. At recruitment, 54% of both female and male patients had stages IIIa-IIIb LC; however, among those remaining at 12 months, most had stages I-II (52% of females, 50% of males), a pattern that persisted beyond 12 months (56% and 57%, respectively). At baseline, adenocarcinoma predominated in females (45%), followed by squamous cell carcinoma (20%), whereas in males, squamous cell carcinoma was most frequent (38%), followed by adenocarcinoma (33%). At 12 months, adenocarcinoma was the most common subtype in both sexes (45% of females, 35% of males). Chemotherapy was used in 55% of females and 49% of males at baseline; among participants with follow-up beyond 12 months, the corresponding proportions were 45% and 39%, respectively. At baseline, SBRT was used in 27% of both sexes; among participants with follow-up beyond 12 months, the corresponding proportions were 47%. IMRT use was 29% in females and 25% in males at baseline, and 35% and 25%, respectively, at 12 months. (Table 2). Radiotherapy was initiated within three months of diagnosis in 75% of females and 67% of males (***Suppl. Table 2***).

**Table 2:** Highest Reported Adverse Events Grade During Follow-up in the REQUITE Lung Cancer Cohort.

		**TOTAL**			**G1+**				**G2+**				**G3+**			
		**Total**	**female**	**male**	**Total**	**female**	**male**		**Total**	**female**	**male**		**Total**	**female**	**male**	
	**Adverse event**	**N**	**N**	**N**	**N (%)**	**N (%)**	**N (%)**	***p***	**N (%)**	**N (%)**	**N (%)**	***p***	**N (%)**	**N (%)**	**N (%)**	***p***
Pulmonary	cough	491	152	339	316 (64.36)	100 (65.79)	216 (63.72)		76 (15.48)	26 (17.11)	50 (14.75)		–	–	–	
	dyspnoea	487	152	335	267 (54.83)	80 (52.63)	187 (55.82)		90 (18.48)	28 (18.42)	62 (18.51)		18 (3.70)	10 (6.58)	8 (2.39)	*
	broncho pulmonary haemorrhage	488	151	337	10 (2.05)	2 (1.32)	8 (2.37)		–	–	–		–	–	–	
	chest wall pain	490	152	338	153 (31.22)	61 (40.13)	92 (27.22)	**	41 (8.37)	21 (13.82)	20 (5.92)	**	7 (1.43)	3 (1.97)	4 (1.18)	
	pneumonitis	485	150	335	114 (23.51)	28 (18.67)	86 (25.67)		44 (9.07)	17 (11.33)	27 (8.06)		3 (0.62)	–	3 (0.89)	
	pulmonary fibrosis	473	142	331	72 (15.22)	21 (14.79)	51 (15.41)		3 (0.63)	–	3 (0.91)		1 (0.21)	–	1 (0.30)	
	bronchial fistula	488	151	337	2 (0.41)	–	2 (0.59)		1 (0.20)	–	1 (0.30)		–	–	–	
	bronchial stricture	488	151	337	51 (10.45)	19 (12.58)	32 (9.50)		36 (7.38)	16 (10.60)	20 (5.93)		1 (0.20)	1 (0.66)	–	
	tracheal fistula	488	151	337	1 (0.20)	–	1 (0.30)		–	–	–		–	–	–	
	tracheal stenosis	488	151	337	1 (0.20)	–	1 (0.30)		–	–	–		–	–	–	
Nervous	brachial plexopathy	488	151	337	9 (1.84)	5 (3.31)	4 (1.19)		4 (0.82)	1 (0.66)	3 (0.89)		1 (0.2)	1 (0.66)	–	
	myelitis	488	151	337	9 (1.84)	5 (3.31)	4 (1.19)		6 (1.23)	4 (2.65)	2 (0.59)		1 (0.2)	1 (0.66)	–	
Oesophageal	oesophagitis	488	151	337	75 (15.37)	30 (19.87)	45 (13.35)		33 (6.76)	17 (11.26)	16 (4.75)	*	5 (1.02)	2 (1.32)	3 (0.89)	
	dysphagia	488	151	337	84 (17.21)	37 (24.50)	47 (13.95)	**	27 (5.53)	12 (7.95)	15 (4.45)		5 (1.02)	2 (1.32)	3 (0.89)	
	oesophageal fistula	488	151	337	1 (0.20)	–	1 (0.30)		–	–	–		–	–	–	
	oesophageal stenosis	488	151	337	8 (1.64)	4 (2.65)	4 (1.19)		5 (1.02)	3 (1.99)	2 (0.59)		1 (0.20)	–	1 (0.30)	
Cardiac	myocardial infarction	488	152	336	7 (1.43)	2 (1.32)	5 (1.49)		7 (1.43)	2 (1.32)	5 (1.49)		4 (0.82)	1 (0.66)	3 (0.89)	
	myocarditis	487	152	335	1 (0.21)	–	1 (0.30)		1 (0.21)	–	1 (0.30)		–	–	–	
	pericardial effusion	488	152	336	11 (2.25)	4 (2.63)	7 (2.08)		11 (2.25)	4 (2.63)	7 (2.08)		1 (0.20)	–	1 (0.30)	
	pericarditis	486	151	335	3 (0.62)	–	3 (0.90)		3 (0.62)	–	3 (0.90)		1 (0.21)	–	1 (0.30)	
Skin	radiation dermatitis	480	151	329	46 (9.58)	15 (9.93)	31 (9.42)		4 (0.83)	2 (1.32)	2 (0.61)		1 (0.21)	–	1 (0.30)	
Maximum	maximum pulmonary adverse event	492	152	340	432 (87.80)	131 (86.18)	301 (88.53)		200 (40.65)	69 (45.39)	131 (38.53)		27 (5.49)	14 (9.21)	13 (3.82)	*
	maximum oesophageal adverse event	488	151	337	119 (24.39)	50 (33.11)	69 (20.47)	**	48 (9.84)	24 (15.89)	24 (7.12)	**	7 (1.43)	2 (1.32)	5 (1.48)	
	maximum overall adverse event	492	152	340	444 (90.24)	135 (88.82)	309 (90.88)		235 (47.76)	80 (52.63)	155 (45.59)		40 (8.13)	16 (10.53)	24 (7.06)	

Abbreviations: N = number of observations; G1 + =any grade of adverse event; G2 + =any grade ≥2 of adverse event; G3 + =any grade ≥3 of adverse event.

*p* = unadjusted *p*-value for the comparison between females and males. ** ≤ 0.01, * ≤ 0.05. Chi-squared test.

**Fig. 1:**
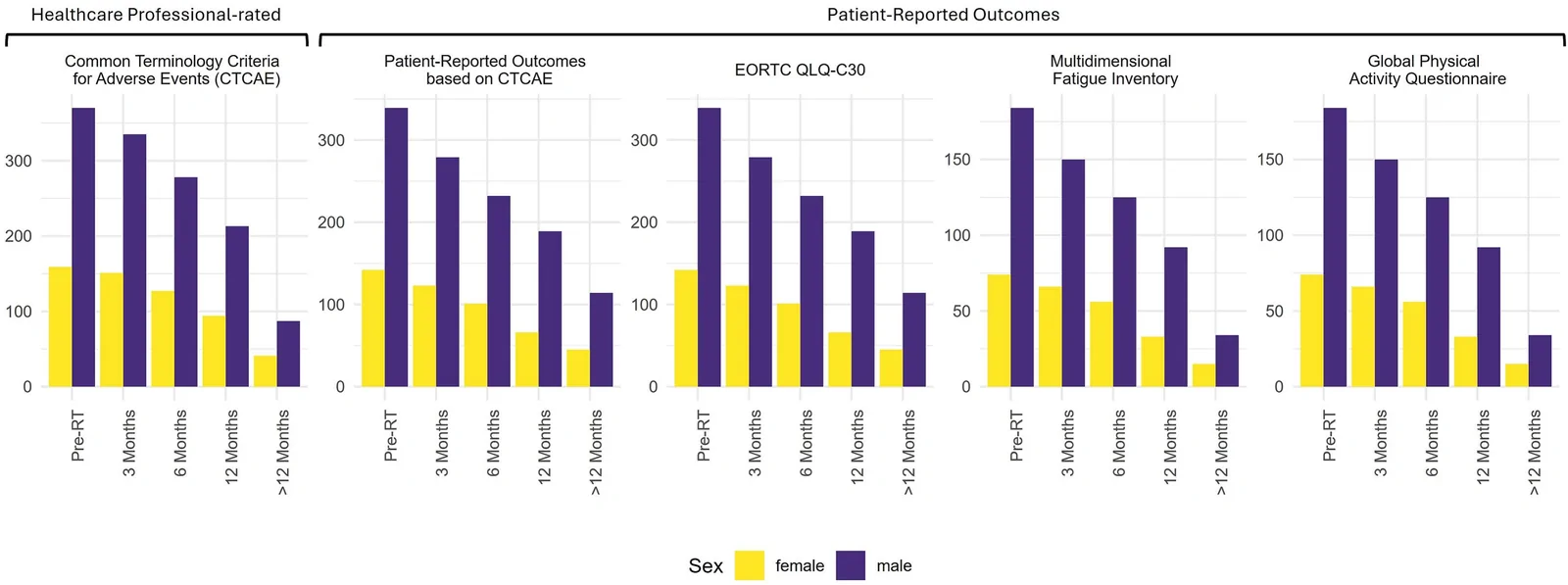
Available Case Report Forms (CRFs) by Timepoint.

At baseline, symptom assessments conducted by healthcare professionals were available for 99.4% of females and 100% of males. PROs, including lung-related symptoms and HRQoL (EORTC QLQ-C30), were provided by 88.8% and 91%, respectively. Physical activity (GPAQ) and fatigue dimensions (MFI) were assessed in 46% of females and 49% of males, as these questionnaires were not implemented at all study sites (Fig. 1***,*** ***Suppl. Table S3***).

At 3, 6, and 12 months, CTCAE-based assessments were available for 94% / 90%, 79% / 75%, and 59% / 57% of females/males, respectively. By 12 months, 32.7% of females and 38.7% of males had died. At 24 months, data availability decreased to 23% for females and 21% for males (Fig. 1***,*** ***Suppl. Table S3***). Vital status and disease progression data were systematically collected up to 12 months post-radiotherapy, with more limited data thereafter (***Suppl. Table S4,*** ***Suppl. Fig. 1***).

### 3.2 Symptom prevalence: Healthcare professional assessments and patient-reported outcomes

**Fig. 2:**
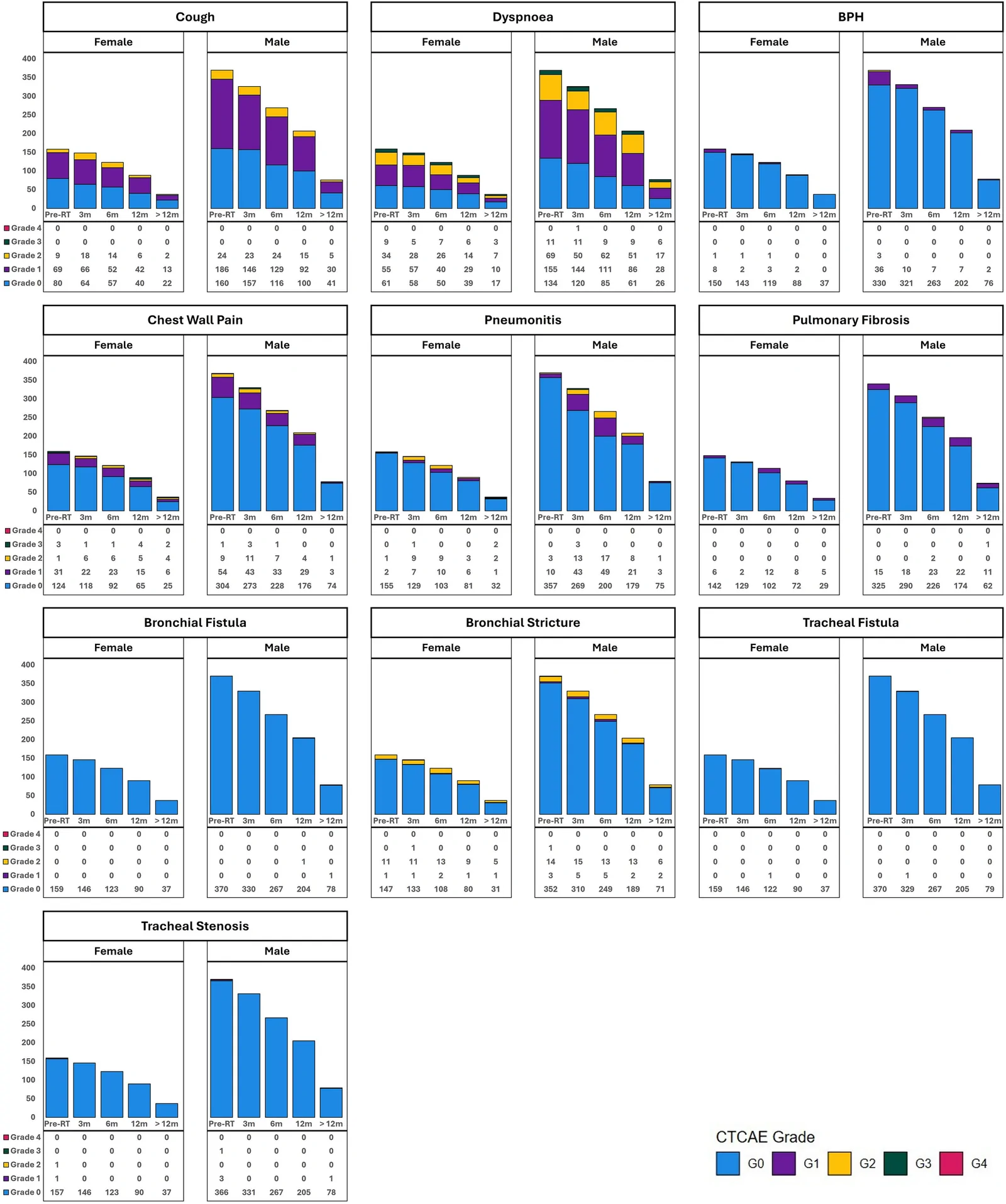

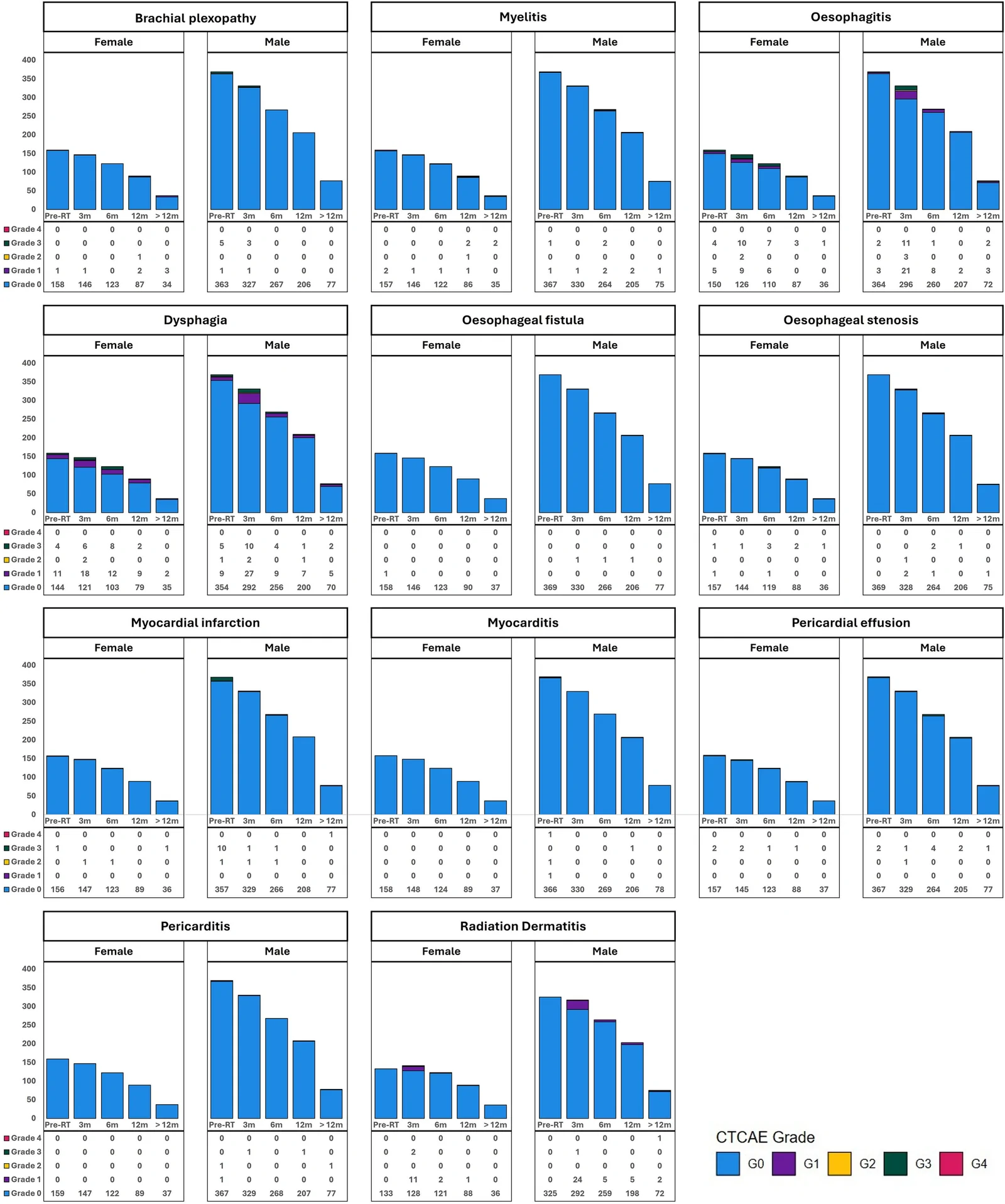
a. Absolute Occurrences of Pulmonary Adverse Events Reported by Healthcare Professionals.

**Fig. 3:**
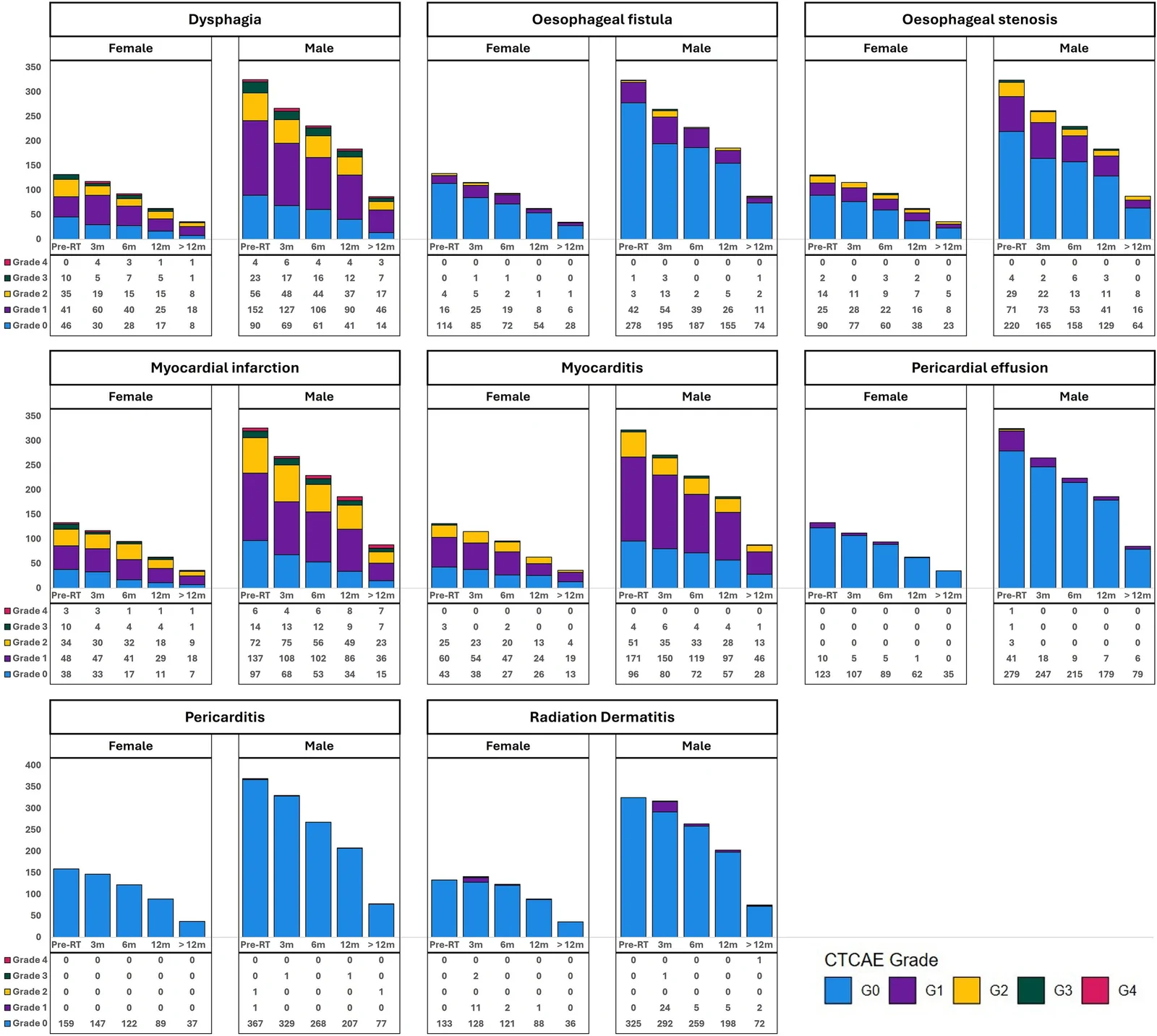
Absolute Occurrences of Pulmonary Adverse Events Reported Through Patient-Reported Outcomes.

Pulmonary symptoms, particularly dyspnoea and cough, were the most frequently reported in both healthcare professionals and patients' reports (see also Aguado-Barrera et al., 2025 [25]). Although adverse events were predominantly reported as grade ≥ 1 throughout follow-up, the vast majority corresponded to CTCAE grades 1–2, while grade ≥ 3 events were infrequent (Fig. 2a***,*** Fig. 3). Oesophageal adverse effects, mainly oesophagitis and dysphagia, was observed primarily at the 3-month follow-up (Fig. 2b).

At 3–6-month post-radiotherapy, 84% of female had grade ≥ 1 adverse events and 46% grade ≥ 2 (***Suppl. Table S5a***); pulmonary grade ≥ 2 events occurred in 40% (***Suppl. Table S5b***) and oesophageal in 15% (***Suppl. Table S5c***). Rates of grade ≥ 2 pulmonary events were similar between SBRT (40%), IMRT (40%) and 3DCRT (38%). Among patients with COPD, 84% experienced grade ≥ 1 and 46% grade ≥ 2 pulmonary adverse events (***Suppl. Table S5b***).

In males, 89% reported grade ≥ 1, and 37% grade ≥ 2 adverse events at 3–6 months (***Suppl. Table S5a***). Pulmonary grade ≥ 2 adverse events occurred in 30% and oesophageal in 6% (***Suppl. Table S5b and S5c***). Grade ≥ 2 pulmonary adverse events were observed in 35% of patients treated with IMRT, 30% of those treated with SBRT, and 23% of the 3DCRT group. COPD remained a key factor (88% grade ≥ 1, 34% grade ≥ 2 pulmonary adverse events) (***Suppl. Table S5b***).

At 12-month, 78% of females had grade ≥ 1 adverse events and 29% grade ≥ 2; among the latter, 35.6% had a pre-existing COPD (***Suppl. Table S5a***). Female treated with SBRT/SABR exhibited the highest incidence of pulmonary grade ≥ 2 symptoms, reaching 35% (***Suppl. Table S5b***).

In males, 81% presented adverse events grade ≥ 1 at 12 months, with grade ≥ 2 reported in 25%. Among male patients with COPD, 40% experienced grade ≥ 2 overall adverse events (***Suppl. Table S5a***), while the highest pulmonary adverse events grade ≥ 2 (37.65%) was observed in patients with COPD (***Suppl. Table S5b).***

Beyond 12 months, 40.5% of females experienced grade ≥ 2 adverse events (***Suppl. Table S5a***). Active smoking and SBRT/SABR were the main contributing factors, with 50% reported grade ≥ 2 adverse events (***Suppl. Table S5a***).

Among male patients with COPD, 42% experienced at least one grade ≥ 2 adverse events beyond 12 months (***Suppl. Table S5a***). This subgroup also had the highest incidence of pulmonary adverse events across all analysed groups (39%; ***Suppl. Table S5b***).

The most impactful patient-reported symptoms were limitations in daily activities, shortness of breath, cough, and feeling tired. In females, the highest frequencies of grade ≥ 1 symptoms or quality-of-life impacts were reduced daily activities and feeling tired beyond 12 months (28/36 and 30/36, respectively), shortness of breath at 12 months (52/63), and cough at 6 months (69/96). In males, the highest rates of grade ≥ 1 symptoms beyond 12 months were impaired daily activities (73/87), shortness of breath (73/88), and feeling tired (74/88). Cough was most frequently reported at 3 months post-radiotherapy (191/271) (Fig. 3).

The quality-of-life data from the EORTC-C30 survey indicated notable differences between female and male patients in physical activity limitations and the impact on family life. In females, 29% reported experiencing “very much” difficulty with strenuous physical activity at baseline, a proportion that remained stable at 12 months post-radiotherapy (30%). Additionally, 6 months after treatment, 15% reported that their family life was interfered with “quite a lot,” and 3% reported interference as “very much.” (***Suppl. Fig. 2***). In males, 14% reported “very much” difficulty with strenuous physical activity at baseline, increasing slightly to 16% at 12 months post-radiotherapy. Regarding family life, 6% reported interference “quite a lot,” and 2% “very much” at 6 months post-treatment (***Suppl. Fig. 2***).

Findings from the MFI questionnaire indicated sex-specific fatigue patterns. At 12 months, 45% of females reported not feeling active at 12 months post-treatment, while 19% indicated generally feeling very active. Beyond 12 months, 47% reported difficulty maintaining physical activity. At 3 months post-radiotherapy, 40% of female patients agreed with the statement, *“I don't want to do anything”* (***Suppl. Fig. 3***)***.*** Among males, 21% reported not feeling active at 12 months, while 16% felt generally very active. After 12 months, 32% reported difficulty staying physically active. At 3 months post-treatment, 59% of male patients agreed with the statement, *“I don't want to do anything”* (***Suppl. Fig. 3***)***.***

### 3.3 Incidence of symptoms

Symptoms incidence during follow-up is detailed in Table 2, with grade trajectories in a Sankey plot in ***Suppl. Fig. 3***. Overall, 40% of patients reported grade ≥ 2 pulmonary adverse event, most commonly dyspnoea (18%) and cough (15%). In females, the most common grade ≥ 2 pulmonary symptoms were dyspnoea (18%) and cough (17%), followed by chest wall pain (13%) and oesophagitis (11%). The most common grade ≥ 3 adverse events included dyspnoea (6%), chest wall pain (3%), oesophagitis (1%) and dysphagia (1%); bronchial stricture, myocardial infarction, and neurological events such as brachial plexopathy and myelitis were present in less than 1%. No grade 4 or 5 events were observed.

In males, dyspnoea (18%) and cough (14%) were also most common. Grade ≥ 2 events exclusive to this group included pulmonary fibrosis, bronchial fistula, myocarditis, and pericarditis. Grade ≥ 3 adverse events observed in males included dyspnoea (2%), pneumonitis (1%), and chest wall pain (1%), with less frequent occurrences (<1%) of pulmonary fibrosis, oesophagitis, dysphagia, oesophageal stenosis, myocardial infarction, pericardial effusion, pericarditis, and radiation dermatitis. Grade 4 adverse events occurred only in males (one acute dyspnoea at three months; two late-onset cardiac events (myocardial infarction and cardiomyopathy) at 24 months); no grade 5 events were reported.

Sex-related differences were observed: females had higher rates of maximum oesophageal events, dysphagia, and chest wall pain at grade ≥ 1 (unadjusted *p* < 0.01), and for oesophagitis, in addition to maximum oesophageal adverse events and chest wall pain at grade ≥ 2, (unadjusted *p* < 0.05). At grade 3, dyspnoea and maximum pulmonary adverse events differed between sexes (unadjusted *p* < 0.05; Table 2).

For acute adverse events, significant sex-related differences (adjusted *p* < 0.05) were observed for overall oesophageal events (grade ≥ 1 and ≥ 2), with the largest differences in the SBRT/SABR, without chemotherapy, clinical stage I-II, non-current smoker, and no-COPD subgroups, and > 70 years (females vs males) (***Suppl. Table S5c***). For late adverse events, sex-related differences were also seen for overall grade ≥ 3 events, primarily driven by pulmonary adverse events in the SBRT/SABR, without chemotherapy and clinical stage I-II subgroups (***Suppl. Table S5b***).

The results of the predefined subgroup analyses are summarized in ***Suppl. Table S6***. Grade ≥ 3 pericarditis and radiation dermatitis were reported exclusively in patients treated with 3DCRT, while grade ≥ 3 pneumonitis and oesophageal stenosis occurred in those receiving IMRT, and grade ≥ 3 pericardial effusion was observed in the SBRT/SABR group (***Suppl. Table S5a***). Chemotherapy increased the incidence of grade ≥ 2 oesophageal adverse events in males from 7.12%in the overall cohort (Table 2) to 11.45% (***Suppl. Table S6b***). Lower frequencies of grade ≥ 2 adverse events were observed among active smokers in both sexes (***Suppl. Table S6c***). In patients with COPD, the prevalence of grade ≥ 2 of dyspnoea, pulmonary fibrosis, bronchial stricture, oesophageal stenosis, pericardial effusion, and pericarditis was increased (***Suppl. Table S6d***). Clinical stage IIIa–IIIb included the only case of grade ≥ 3 pericarditis (***Suppl. Table S6e***).

In patients >70 years, grade ≥ 2 oesophagitis decreased in both sexes (females: 12% to 8%, males: 8% to 0.6%), with no myocardial infarctions or grade ≥ 2 pericardial effusions observed in females (***Suppl. Table S6f***). Significant sex-related differences (adjusted *p* < 0.05) were seen in maximum pulmonary adverse events after SBRT/SABR, with higher incidence in females. Females also showed higher rates of maximum overall adverse events in the >70-year group The most pronounced sex differences across adverse events and grades were observed in clinical stage I–II, with consistently higher proportions in females (***Suppl. Table S6***).

## 4 Discussion

This study provides a prospective, multicentre analysis of 530 LC patients undergoing radiotherapy, with systematically collected clinical, dosimetric, adverse events, and patient-reported outcome data. The principal strength of this cohort lies in its standardized longitudinal data capture across multiple European and US centres, enabling detailed characterisation of treatment-related adverse events in a real-world setting. While the heterogeneity of disease stage and treatment modalities limits formal comparative analyses, the dataset provides a robust resource for benchmarking and hypothesis generation.

Over time, the composition of the study cohort shifted, with a higher proportion of patients with early-stage disease (stage I–II) remaining under observation after 12 months, reflecting the better prognosis of this group. This trend is consistent with patterns commonly observed in long-term observational studies of LC, where disease progression and associated morbidity often limit extended follow-up in patients with advanced-stage tumours [26].

Pulmonary adverse events, consistent with previous studies [27], were the most frequently reported by both healthcare professionals and patients. Dyspnoea and cough were the predominant symptoms, particularly among patients with pre-existing COPD, as expected. However, it is important to note that the occurrence of such symptoms cannot be automatically labelled as radiotherapy-related adverse events due to the prevalence of comorbidities. At the three-month follow-up, a higher proportion of male patients (89%) experienced any grade ≥ 1 adverse events compared to female patients (85%). However, a greater proportion of female patients reported grade ≥ 2 symptoms, consistent with findings from Radiation Therapy Oncology Group (RTOG) trials, which demonstrated higher adverse event rates among women [28]. SBRT was associated with lower rates of overall acute grade ≥ 2 adverse events, reflecting earlier disease stage and smaller tumour volume at diagnosis [29]. Additionally, sex specific characteristics in quality of life were observed, with female patients reporting greater difficulties in performing physical activities following treatment. These findings warrant further investigation, as previous studies suggest that multiple factors may influence this perception [30].

In this study, we present a descriptive analysis from a sex-based perspective. Although female patients are underrepresented in the cohort, this distribution reflects global incidence trends in LC [31]. Sex-specific characteristics in LC affect various aspects of the disease including diagnosis, histological subtype, treatment response, adverse event profiles, and survival outcomes [32], [33], [34], [35]. Women are more frequently diagnosed at earlier stages, are more likely to be never-smokers, and more commonly present with adenocarcinoma histology [36], [37], [38], [39]. While women generally show better overall outcomes [40], this survival advantage may diminish in advanced stages of disease [41] in specific histological subtypes [42] or among patients who continue to smoke during radiotherapy [43]. Consistent with previous reports, women in our cohort experience higher rates of grade ≥ 2 and grade ≥ 3 treatment-related adverse events [35], [44]. However, the few grade 4 events observed occurred exclusively in men, primarily involving cardiac events. Given the very small number of such events and the prevalence of baseline comorbidities, this observation should be interpreted with caution and cannot be considered evidence of a sex-specific effect. Men also typically exhibit poorer overall and disease-free survival, which may be partly attributable by sex-specific differences in the impact of treatment-related factors, including responses to chemotherapy, surgery, or the sequencing of therapies [32], [45]. These differences may also reflect variations in lifestyle-related factors, although these were not specifically investigated in the present study. Although limited sex-specific data are available on radiotherapy outcomes [46], sex is frequently included as an analytical variable in clinical studies. Some evidence suggests that women may derive greater benefit from radiotherapy than men [47], [48], [49]. Considering these disparities, we incorporated a sex-based descriptive analysis, in line with growing recognition of their importance in advancing personalized LC care [28], [50], [51], [52]. Our results suggest that the observed patterns may reflect sex-related differences in adverse events profiles, particularly among patients treated with Clinical Stage I-II, SBRT/SABR and those aged >70 years. However, given the observational design and limited subgroup sizes, these results should be considered hypothesis-generating.

One of the primary objectives of REQUITE was to assess the incidence of radiotherapy-related adverse events at 12 months in LC patients. Although some centres provided extended follow-up, reduced data availability beyond this time point was anticipated given overall survival rates in LC [53], as well as practical constraints such as limited funding, institutional follow-up policies, and the suspension of in-person visits during the COVID-19 pandemic (2020−2022). In addition, the lower availability of MFI and GPAQ data was partly due to these questionnaires being implemented in only 11 of the 16 participating centres. Finally, the lack of data on vital status, recurrence, and disease progression after 12 months limits survival analyses and assessment of tumour response.

While REQUITE remains one of the most comprehensive multicentre cohorts in this setting, with a satisfactory recruitment rate, the overall sample size limits the statistical power of subgroup analyses, particularly after stratification by radiotherapy modality or by clinical, dosimetric, or pathological variables. Nevertheless, the cohort is sufficiently powered to serve as a validation dataset and to contribute meaningfully to future pooled analyses and meta-analyses [54], [55], [56]. Although patient inclusion was completed in 2017, the biological relevance of this cohort extends beyond specific radiotherapy techniques. Determinants of normal tissue radiosensitivity, including germline genetic variants and circulating biomarkers, are not expected to be substantially affected by changes in dose delivery technologies. Therefore, this dataset remains highly relevant for biomarker validation and the development of predictive models, particularly when integrated with contemporary datasets.

In summary, the REQUITE-Lung cohort provides a structured real-world resource for characterising radiotherapy-related adverse events and patient-reported outcomes in lung cancer. Its main value lies in supporting external validation, pooled analyses, and translational research, rather than in direct comparative assessment of treatment modalities. These data provide a foundation for future studies aimed at refining risk stratification and improving personalized radiotherapy strategies.

## Funding

REQUITE was funded from the European Union's Seventh Framework Programme for research, technological development and demonstration under grant agreement #601826.

Contract grant sponsor Ana Vega: supported by Spanish Instituto de Salud Carlos III (ISCIII) funding, an initiative of the Spanish Ministry of Economy and Innovation partially supported by European Regional Development FEDER Funds (PI25/00744, PI22/00589, PI19/01424; INT24/00023; DTS24/00083); the ERAPerMed JTC2018 funding (AC18/00117); the Autonomous Government of Galicia (Consolidation and structuring program: IN607B2025/009), and by the AECC (PRYES211091VEGA). Sara Gutiérrez-Enríquez was supported by ERAPerMed JTC2018 funding (ERAPERMED2018–244 and SLT011/18/00005) and the 10.13039/501100002809Generalitat de Catalunya (2021SGR01112). Corinne Faivre-Finn is funded by the National Institute for Health and Care Research (NIHR) Manchester Biomedical Research Centre (BRC) (NIHR203308).

*Abbreviations:* EORTC QLQ-C30 = European Organization for Research and Treatment for Cancer quality of life questionnaire; pre-RT = pre-radiotherapy

*Abbreviations:* BPH=bronchopulmonary haemorrhage; N = number of observations; pre-RT = pre-radiotherapy; G = grade**.** Fig. 2b. Absolute Occurrences of Neurological, Oesophageal, Cardiac, and Dermatological Adverse Events Reported by Healthcare Professionals.

*Abbreviations:* N = number of observations; pre-RT = pre-radiotherapy; G = grade

*Abbreviations:* N = number of observations; pre-RT = pre-radiotherapy; G = grade.

## Declaration of competing interest

The authors declare the following financial interests/personal relationships which may be considered as potential competing interests :Co-author D. Azria was involved in the creation of the start-up NovaGray in 2015. co-author D. De Ruysscher: none related to the current manuscript. Outside the current manuscript: advisory board of Astra Zeneca, Bristol-Myers-Squibb, Roche/Genentech, Merck/Pfizer, Celgene, Noxxon, Mologen and has received investigator-initiated grants from Bristol-Myers-Squibb, Boehringer Ingelheim and Astra-Zeneca. co-author E. Sperk: none related to the current manuscript. Outside the current manuscript: General speakers bureau Zeiss Meditec, travel support Zeiss Meditec. The other authors declare that they do not have any conflict of interest. If there are other authors, they declare that they have no known competing financial interests or personal relationships that could have appeared to influence the work reported in this paper.
