# Supplementary material for: Early and late CTCAE and patient-reported outcomes following lung cancer radiotherapy: A sex-stratified descriptive analysis from the REQUITE cohort
Source: Clin Transl Radiat Oncol. 2026 Aug 20;61:101265. doi: 10.1016/j.ctro.2026.101265 (PMC13543982; doi:10.1016/j.ctro.2026.101265)

SUPPLEMENTARY MATERIAL

Index of Supplementary Methods

- ***Supplementary Methods.*** Patient Data Collection 2

Index of Supplementary Tables

- ***Supplementary Table S1.*** Additional Baseline Characteristics of REQUITE Lung Cancer Patients 3
- ***Supplementary Table S2.*** Time From Diagnosis to Treatment and Duration of Treatment 4
- ***Supplementary Table S3.*** Data Available for the REQUITE Lung Cancer Cohort, Categorized by Case Report Form (CRF) and Timepoint 5
- ***Supplementary Table S4.*** Vital Status Reported at 12 Months in the REQUITE Lung Cancer Cohort 6

Index of Supplementary Figures

- ***Supplementary Figure 1.*** Sex-Stratified Kaplan–Meier Curves in Lung Cancer Patients treated with Radiotherapy 7
- ***Supplementary Figure 2.*** Relative Frequencies of Patient-Reported Health-Related Quality of Life Outcomes Using the EORTC QLQ-C30 Questionnaire 8
- ***Supplementary Figure 3.*** Relative Frequencies of Patient-Reported Outcomes from the Multidimensional Fatigue Inventory (MFI) 10
- ***Supplementary Figure 4.*** Sex-Stratified Sankey Representation of CTCAE Grades of Adverse Events from Baseline to Late Follow-up in Lung Cancer Patients treated with Radiotherapy 11

Supplementary Data Files

- ***Supplementary Table S5.xlsx***
  - ***S5a.*** Cumulative Incidence of Acute and Late Overall Adverse Events
  - ***S5b.*** Cumulative Incidence of Acute and Late Pulmonary Adverse Events
  - ***S5c.*** Cumulative Incidence of Acute and Late Oesophageal Adverse Events

- ***Supplementary Table S6.xlsx***
  - ***S6a.*** Sex-specific maximum toxicity rates following radiotherapy, adjusted for baseline values and stratified by radiotherapy technique
  - ***S6b.*** Sex-specific maximum toxicity rates following radiotherapy, adjusted for baseline values and stratified by chemotherapy use
  - ***S6c.*** Sex-specific maximum toxicity rates following radiotherapy, adjusted for baseline values and stratified by smoking status
  - ***S6d.*** Sex-specific maximum toxicity rates following radiotherapy, adjusted for baseline values and stratified by chronic obstructive pulmonary disease
  - ***S6e.*** Sex-specific maximum toxicity rates following radiotherapy, adjusted for baseline values and stratified by clinical stage
  - ***S6f.*** Sex-specific maximum toxicity rates following radiotherapy, adjusted for baseline values and stratified by age

Supplementary Methods.

Patient Data Collection

Data were collected prospectively for at least one year after radiotherapy using standardized methods [1], with a focus on radiotherapy-related adverse events, non-genetic factors (e.g., demographics, co-morbidities, treatments, radiotherapy dosimetry, chemotherapy, pulmonary function tests), and quality of life. Information was recorded using case report forms and stored in a centralized electronic database. Assessments were conducted prior to the start of radiotherapy and at 3, 6, and 12 months, with optional extended follow-up conducted annually thereafter.

Healthcare professionals assessed 21 symptoms using the Common Terminology Criteria for Adverse Events (CTCAE v.4.0) [2], categorizing them by the organ at risk. These included pulmonary adverse events such as cough, dyspnoea, broncho-pulmonary haemorrhage, chest wall pain, pneumonitis, pulmonary fibrosis, bronchial fistula, bronchial stricture, tracheal fistula, and tracheal stenosis; nervous adverse events, including brachial plexopathy and myelitis; oesophageal toxicities, such as oesophagitis, dysphagia, oesophageal fistula, and oesophageal stenosis; cardiac events —including myocardial infarction, myocarditis, pericardial effusion, and pericarditis— and radiation dermatitis for skin adverse event.

Participants completed the REQUITE Patient Questionnaires—CTCAE-based Patient Report Outcomes (PRO) [3] adapted into a lay language for patients with lung cancer to assess symptoms related to radiation-induced adverse effects. In addition, PROs were used to evaluate health-related QoL (HRQoL) using the European Organization for Research and Treatment for Cancer Quality of Life Questionnaire (EORTC QLQ-C30) [4], fatigue dimensions via the Multidimensional Fatigue Inventory (MFI) [5] and physical activity using the Global Physical Activity Questionnaire (GPAQ) [6]. Centres administered questionnaires in paper or electronic format, according to local protocols and patient requirements. For global analyses, PRO and HRQoL data were included if at least one item was completed.

All comprehensive questionnaire data were linked to additional available information and the biorepository, including relevant clinical metadata (e.g., DICOM-RT files), genotyping data (*Infinium OncoArray-500K BeadChip, Illumina*), a biobank of biological samples (DNA and *PAXgene Blood RNA* tubes), and results from the radiation-induced lymphocyte apoptosis (RILA) assay [7].

Additional lung cancer cohorts were included in the REQUITE project, comprising 282 patients from the UK-CONVERT trial (Concurrent Once-Daily Versus Twice-Daily Chemoradiotherapy in Patients with Limited-Stage Small-Cell Lung Cancer) [8], 82 patients from Ghent University, and 19 UK patients from the RADAR study (FLT PET in Radiation Damage and Resistance in Lung Cancer Radiotherapy) [9]. Although these data were not included in the present extended analysis, sample and genotype information, as well as partial datasets, are available for reference.

References:

[1] Seibold P, Webb A, Aguado-Barrera ME, Azria D, Bourgier C, Brengues M, et al. REQUITE: A prospective multicentre cohort study of patients undergoing radiotherapy for breast, lung or prostate cancer. Radiotherapy and Oncology 2019;138. https://doi.org/10.1016/j.radonc.2019.04.034.

[2] National Cancer Institute. Common Terminology Criteria for Adverse Events (CTCAE) version 4.0. NIH Publication 2009. https://doi.org/10.1080/00140139.2010.489653.

[3] Christodoulou M, McCloskey P, Stones N, Bayman N, Burt P, Chittalia A, et al. Investigation of a Patient Reported Outcome tool to assess radiotherapy-related toxicity prospectively in patients with lung cancer. Radiotherapy and Oncology 2014;112:244–9. https://doi.org/10.1016/J.RADONC.2014.07.008.

[4] Aaronson NK, Ahmedzai S, Bergman B, Bullinger M, Cull A, Duez NJ, et al. The European organization for research and treatment of cancer QLQ-C30: A quality-of-life instrument for use in international clinical trials in oncology. J Natl Cancer Inst 1993. https://doi.org/10.1093/jnci/85.5.365.

[5] Smets EMA, Garssen B, Bonke B, De Haes JCJM. The multidimensional Fatigue Inventory (MFI) psychometric qualities of an instrument to assess fatigue. J Psychosom Res 1995;39. https://doi.org/10.1016/0022-3999(94)00125-O.

[6] Bull FC, Maslin TS, Armstrong T. Global physical activity questionnaire (GPAQ): Nine country reliability and validity study. J Phys Act Health 2009;6. https://doi.org/10.1123/jpah.6.6.790.

[7] Talbot CJ, Veldwijk MR, Azria D, Batini C, Bierbaum M, Brengues M, et al. Multi-centre technical evaluation of the radiation-induced lymphocyte apoptosis assay as a predictive test for radiotherapy toxicity. Clin Transl Radiat Oncol 2019;18. https://doi.org/10.1016/j.ctro.2019.06.001.

[8] Faivre-Finn C, Snee M, Ashcroft L, Appel W, Barlesi F, Bhatnagar A, et al. Concurrent once-daily versus twice-daily chemoradiotherapy in patients with limited-stage small-cell lung cancer (CONVERT): an open-label, phase 3, randomised, superiority trial. Lancet Oncol 2017. https://doi.org/10.1016/S1470-2045(17)30318-2.

[9] Koh PK, Dean E, Trigonis I, Jackson A, Faivre-Finn C, Blackhall FH. RADAR Radiation damage and resistance in patients with lung cancer. Lung Cancer 2010;67:S32–3. https://doi.org/10.1016/s0169-5002(10)70100-8.

Supplementary Table S1.

Additional Baseline Characteristics of REQUITE Lung Cancer Patients

	Pre-RT (N=530)		12 months Post-RT (N=310)		> 12 months Post-RT (N=151)	
	female (N=159) N (%)	male (N=371) N (%)	female (N=91) N (%)	male (N=219) N (%)	female (N=48) N (%)	male (N=103) N (%)
**Alcohol intake**						
never	62 (39)	101 (27.2)	30 (33)	52 (23.7)	14 (29.2)	14 (13.6)
former	24 (15.1)	54 (14.6)	14 (15.4)	31 (14.2)	7 (14.6)	14 (13.6)
current	61 (38.4)	181 (48.8)	39 (42.9)	113 (51.6)	22 (45.8)	61 (59.2)
missing	12 (7.5)	35 (9.4)	8 (8.8)	23 (10.5)	5 (10.4)	14 (13.6)
**Menopausal status**						
pre	5 (3.1)	-	2 (2.2)	-	3 (6.3)	-
post	140 (88.1)	-	80 (87.9)	-	39 (81.3)	-
peri	4 (2.5)	-	3 (3.3)	-	2 (4.2)	-
missing	10 (6.3)	-	6 (6.6)	-	4 (8.3)	-
**Diabetes or use of antidiabetic**						
yes	19 (11.9)	69 (18.6)	11 (12.1)	38 (17.4)	4 (8.3)	18 (17.5)
**Hypertension or use of antihypertensives**						
yes	79 (49.7)	222 (59.8)	47 (51.6)	136 (62.1)	22 (45.8)	65 (63.1)
**History of heart disease or use of amiodarone**						
yes	34 (21.4)	131 (35.3)	19 (20.9)	77 (35.2)	7 (14.6)	37 (35.9)
**Rheumatoid arthritis**						
yes	8 (5)	9 (2.4)	7 (7.7)	7 (3.2)	3 (6.3)	2 (1.9)
**Systemic lupus erythematosus**						
no	159 (100)	370 (99.7)	91 (100)	218 (99.5)	48 (100)	103 (100)
**Collagen vascular diseases**						
yes	5 (3.1)	13 (3.5)	5 (5.5)	9 (4.1)	2 (4.2)	5 (4.9)
**Depression or use of antidepressant**						
yes	44 (27.7)	47 (12.7)	25 (27.5)	29 (13.2)	14 (29.2)	17 (16.5)
**Tuberculosis**						
yes	3 (1.9)	10 (2.7)	3 (3.3)	6 (2.7)	-	2 (1.9)
**On statin or other lipid lowering drugs**						
yes	65 (40.9)	157 (42.3)	36 (39.6)	95 (43.4)	16 (33.3)	49 (47.6)
**On oral steroids**						
yes	12 (7.5)	24 (6.5)	5 (5.5)	6 (2.7)	2 (4.2)	3 (2.9)
**On analgesics**						
yes	41 (25.8)	68 (18.3)	24 (26.4)	39 (17.8)	11 (22.9)	20 (19.4)
**On immunosuppressant**						
yes	4 (2.5)	4 (1.1)	1 (1.1)	1 (0.5)	1 (2.1)	-
**First-degree family history of lung cancer**						
yes	29 (18.2)	66 (17.8)	16 (17.6)	35 (16)	8 (16.7)	21 (20.4)
**Tumour primary side**						
RUL	48 (30.2)	131 (35.4)	33 (36.3)	76 (34.7)	20 (41.7)	34 (33.0)
RML	8 (5.0)	17 (4.6)	2 (2.2)	7 (3.2)	1 (2.1)	2 (1.9)
RLL	25 (15.7)	48 (13.0)	8 (8.8)	35 (16.0)	5 (10.4)	14 (13.6)
R. Lung	11 (6.9)	16 (4.3)	6 (6.6)	11 (5.0)	2 (4.2)	5 (4.9)
LUL	42 (26.4)	93 (25.1)	26 (28.6)	54 (24.7)	12 (25.0)	30 (29.1)
Lingular	1 (0.6)	2 (0.5)	1 (1.1)	1 (0.5)	1 (2.1)	0 (0)
LLL	22 (13.8)	46 (12.4)	14 (15.4)	24 (11.0)	7 (14.6)	13 (12.6)
L. Lung	1 (0.6)	16 (4.3)	1 (1.1)	10 (4.6)	0 (0)	4 (3.9)
missing	1 (0.6)	1 (0.3)	0 (0)	1 (0.5)	0 (0)	1 (1.0)
**Histology**						
Squamous	33 (20.8)	142 (38.4)	14 (15.4)	77 (35.2)	4 (8.3)	32 (31.1)
Adenocarcinoma	72 (45.3)	122 (33.0)	40 (44.0)	71 (32.4)	22 (45.8)	36 (35.0)
Large Cell	2 (1.3)	18 (4.9)	1 (1.1)	11 (5.0)	0 (0)	4 (3.9)
Mixed	4 (2.5)	7 (1.9)	2 (2.2)	4 (1.8)	3 (6.3)	3 (2.9)
Small Cell	35 (22.0)	65 (17.6)	27 (29.7)	44 (20.1)	15 (31.3)	24 (23.3)
Other	10 (6.3)	10 (2.7)	5 (5.5)	7 (3.2)	2 (4.2)	4 (3.9)
missing	3 (1.9)	6 (1.6)	2 (2.2)	5 (2.3)	2 (4.2)	0 (0)
**Clinical Tumour stage**						
0	1 (0.63)	9 (2.43)	1 (1.1)	7 (3.2)	1 (2.08)	5 (4.85)
1a, 1b	59 (37.11)	120 (32.43)	40 (43.96)	85 (38.81)	28 (58.33)	45 (43.69)
2a, 2b	44 (27.67)	96 (25.95)	21 (23.08)	54 (24.66)	5 (10.42)	22 (21.36)
3, 4	53 (33.33)	139 (37.57)	29 (31.87)	69 (31.51)	14 (29.17)	29 (28.16)
missing	2 (1.26)	6 (1.62)	0 (0)	4 (1.83)	0 (0)	2 (1.94)
**Clinical Lymph Nodes stage**						
0	69 (43.4)	166 (44.9)	47 (51.6)	111 (50.7)	28 (58.3)	62 (60.2)
1	12 (7.5)	36 (9.7)	7 (7.7)	23 (10.5)	2 (4.2)	7 (6.8)
2	50 (31.4)	123 (33.2)	28 (30.8)	67 (30.6)	15 (31.3)	28 (27.2)
3	27 (17.0)	39 (10.5)	9 (9.9)	14 (6.4)	3 (6.3)	4 (3.9)
missing	1 (0.6)	6 (1.6)	0 (0)	4 (1.8)	0 (0)	2 (1.9)
**Surgical intervention**						
yes	17 (10.7)	32 (8.6)	7 (7.7)	22 (10.0)	3 (6.3)	10 (9.7)
**Radiotherapy interrupted**						
yes	6 (3.8)	26 (7.0)	2 (2.2)	16 (7.3)	1 (2.1)	9 (8.7)
missing	1 (0.6)	0 (0)	1 (1.1)	0 (0)	0 (0)	0 (0)
**Total dose (Gy)**						
Range [min – max]	[8.58 - 70.0]	[19.3 - 117]	[45.0 - 70.0]	[45.0 - 80.0]	[45.0 - 70.0]	[45.0 - 75.7]
**Radiotherapy regimens**						
Conventional fractionation/moderate hypofractionation						
1.5Gy per fx, 30-42 fx	29 (7.8)	19 (11.9)	17 (7.8)	9 (9.8)	12 (11.7)	6 (12.5)
1.8-1.99Gy per fx, 25-39 fx	24 (6.5)	6 (3.8)	6 (2.8)	2 (2.2)	1 (1.0)	1 (2.1)
2.0Gy per fx, 21-40 fx	122 (33.0)	58 (36.3)	64 (29.4)	28 (30.4)	23 (22.3)	14 (29.2)
>2.0-3.0Gy per fx, 20-33 fx	80 (21.6)	29 (18.1)	43 (19.7)	14 (15.2)	17 (16.5)	4 (8.3)
4.0-5.0 Gy per fx, 12-24 fx	12 (3.2)	4 (2.5)	7 (3.2)	3 (3.3)	1 (1.0)	0 (0)
Stereotactic body radiotherapy						
18-20Gy per fx, 3 fx	23 (6.2)	7 (4.4)	17 (7.8)	6 (6.5)	9 (8.7)	3 (6.3)
8.75-15Gy per fx, 4 fx	45 (12.2)	19 (11.9)	35 (16.1)	18 (19.6)	24 (23.3)	12 (25)
10-15.27Gy per fx, 5 fx	17 (4.6)	13 (8.1)	15 (6.9)	7 (7.6)	7 (6.8)	4 (8.3)
7.5-9.18Gy per fx, 8 fx	18 (4.9)	5 (3.1)	14 (6.4)	5 (5.4)	9 (8.7)	4 (8.3)
**Mean lung dose (Gy)**						
median (IQR)	10.4 (11.2)	10.9 (10.6)	9.32 (9.2)	9.76 (11.4)	8.27 (9.4)	9.04 (11.1)
missing	16 (10.1)	31 (8.4)	5 (5.5)	21 (9.6)	4 (8.3)	10 (9.7)
**V20 lung (%)**						
median (IQR)	16.2 (20.1)	16.4 (19.0)	14.1 (17.7)	14.7 (18.5)	12.6 (16.4)	13.3 (17.8)
missing	6 (3.8)	5 (1.4)	2 (2.2)	2 (0.9)	2 (4.2)	1 (1.0)
**V35 oesophagus (%)**						
median (IQR)	20.4 (37.1)	18.5 (35)	17.2 (34.4)	16.1 (31.5)	15.4 (33.6)	12.1 (24.0)
missing	7 (4.4)	11 (3.0)	1 (1.1)	5 (2.3)	1 (2.1)	2 (1.9)

Abbreviations: Pre-RT=Pre-Radiotherapy; m=months; Post-RT=post-Radiotherapy; N=number of observations; RUL=right upper lobe; RML=right middle lobe; RLL=right lower lobe; R=right; LUL=left upper lobe; LLL=left lower lobe; L=left; 3D CRT=Three Dimensional (3D) Conformal Radiation Therapy; VMAT= Volumetric Modulated Arc Therapy; IMRT= Intensity-Modulated Radiotherapy; min=minimum value; max=maximum value; fx=fraction; IQR=interquartile range; V20=volume receiving ?20 Gy; V35=volume receiving ? 35 Gy.

Supplemenary Table 2.

Time from Diagnosis to Treatments and Treatments Duration.

	Time to Surgery		Time to Chemotherapy		Chemotherapy Duration		Time to Radiotherapy		Radiotherapy Duration	
	female (N=17)	male (N=32)	female (N=86)	male (N=182)	female (N=86)	male (N=179)	female (N=154)	male (N=361)	female (N=159)	male (N=371)
**0-1 month**	9 (52.9)	16 (50)	43 (50)	89 (48.9)	7 (8.1)	21 (11.7)	33 (21.4)	48 (13.3)	84 (52.8)	177 (47.7)
**1-3 months**	2 (11.8)	9 (28.1)	39 (45.3)	82 (45.1)	70 (81.4)	130 (72.6)	83 (53.9)	197 (54.6)	75 (47.2)	194 (52.3)
**3-6 months**	6 (35.3)	6 (18.8)	2 (2.3)	6 (3.3)	8 (9.3)	22 (12.3)	26 (16.9)	83 (23)	-	-
**6-12 months**	-	-	2 (2.3)	2 (1.1)	1 (1.2)	5 (2.8)	10 (6.5)	21 (5.8)	-	-
**>12 months**	-	1 (3.1)	-	3 (1.6)	-	1 (0.6)	2 (1.3)	12 (3.3)	-	-

Time to Surgery: Number of months from cancer diagnosis to surgery.
Time to Chemotherapy: Number of months from cancer diagnosis to the start of chemotherapy.
Chemotherapy Duration: Total duration of chemotherapy.
Time to Radiotherapy: Number of months from cancer diagnosis to the start of radiotherapy.
Radiotherapy Duration: Total duration of radiotherapy.

Supplementary Table S3.

Data Available for the REQUITE Lung Cancer Cohort, Categorized by Case Report Form (CRF) and Timepoint

	Pre-RT		3 months post-RT		6 months post-RT		12 months post-RT		24 months post-RT		36 months post-RT		48 months post-RT		60 months post-RT		72 months post-RT	
**CRF**	**female**	**male**	**female**	**male**	**female**	**male**	**female**	**male**	**female**	**male**	**female**	**male**	**female**	**male**	**female**	**male**	**female**	**male**
	N	N	N (%)	N (%)	N (%)	N (%)	N (%)	N (%)	N (%)	N (%)	N (%)	N (%)	N (%)	N (%)	N (%)	N (%)	N (%)	N (%)
L2-Patient factors	160	370	151 (94.38)	337 (91.08)	127 (79.38)	275 (74.32)	91 (56.88)	207 (55.95)	38 (23.75)	78 (21.08)	3 (1.88)	6 (1.62)	-	-	-	-	0	3 (0.81)
L3-Clinical and Treatment data *	-	-	160	370	-	-	-	-	-	-	-	-	-	-	-	-	-	-
L4-Health Professional Lung Toxicity data	159	370	151 (94.97)	335 (90.54)	127 (79.87)	278 (75.14)	94 (59.12)	213 (57.57)	37 (23.27)	79 (21.35)	3 (1.89)	6 (1.62)	-	-	-	-	1 (0.63)	2 (0.54)
PRO-Lung Symptoms questionaire	142	339	123 (86.62)	279 (82.30)	101 (71.13)	232 (68.44)	66 (46.48)	189 (55.75)	31 (21.83)	76 (22.42)	4 (2.82)	16 (4.72)	6 (4.23)	11 (3.24)	3 (2.11)	9 (2.65)	1 (0.70)	2 (0.59)
PRO-EORTC QLQ-C30	142	339	123 (86.62)	279 (82.30)	101 (71.13)	232 (68.44)	66 (46.48)	189 (55.75)	31 (21.83)	76 (22.42)	4 (2.82)	16 (4.72)	6 (4.23)	11 (3.24)	3 (2.11)	9 (2.65)	1 (0.70)	2 (0.59)
PRO-GPAQ	74	184	66 (89.19)	150 (81.52)	56 (75.68)	125 (67.93)	33 (44.59)	92 (50.00)	14 (18.92)	28 (15.22)	1 (1.35)	5 (2.72)	-	-	-	-	0	1 (0.54)
PRO-MFI	74	184	66 (89.19)	150 (81.52)	56 (75.68)	125 (67.93)	33 (44.59)	92 (50.00)	14 (18.92)	28 (15.22)	1 (1.35)	5 (2.72)	-	-	-	-	0	1 (0.54)

Abbreviations: PRO=Patient Report Outcomes; EORTC QLQ-C30=European Organisation for Research and Treatment of Cancer Quality of Life Questionnaire Core 30; GPAQ=Global Physical Activity Questionnaire; MFI=Multidimensional Fatigue Inventory; Pre-RT=Pre-Radiotherapy

% Percentage calculated based on the total number of baseline questionnaires for each sex

* Data for this questionnaire were collected only at the third-month follow-up

Note: The reduction in data beyond the 12-month follow-up is due to non-participation of some recruitment centers in the extended follow-up, disease progression or death, and loss to follow-up related to the COVID-19 pandemic.

Supplementary Table S4.

Vital status reported at 12 months in the REQUITE lung cohort.

Total			Death by any cause		Withdrawals		Without recurrence		Local recurrence		Distant recurrence		Second cancer	
**Total**	female	male	female	male	female	male	female	male	female	male	female	male	female	male
**N**	N (%)*	N (%)*	N (%)	N (%)	N (%)	N (%)	N (%)	N (%)	N (%)	N (%)	N (%)	N (%)	N (%)	N (%)
**515**	156 (30.29)	359 (69.71)	51 (32.69)	139 (38.72)	46 (29.49)	93 (25.91)	64 (41.03)	135 (37.6)	45 (28.85)	107 (29.81)	38 (24.36)	81 (22.56)	5 (3.21)	12 (3.34)

The included frequencies refer to the number of events ever recorded for each category, without being necessarily mutually exclusive between one category and the other (i.e., the same subject can contribute with data for more than one category)

* % Calculated as a percentage of the total number of individuals

% Calculated as a percentage of the total number of individuals for each sex

Supplementary Figure 1. Sex-Stratified Kaplan–Meier Curves in Lung Cancer Patients treated with Radiotherapy

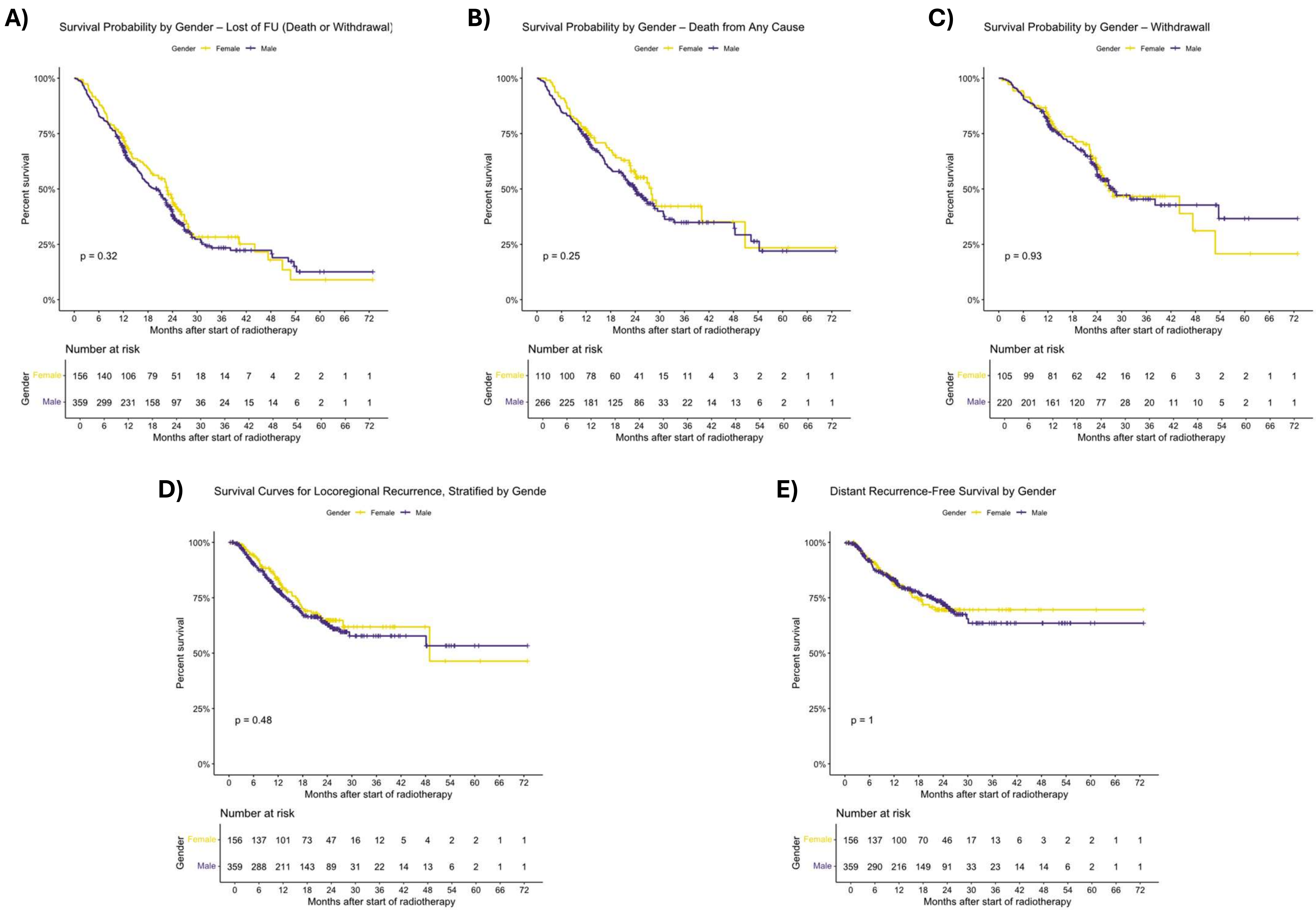

Supplementary Figure 2. Relative Frequencies of Health-Related Quality of Life (HRQoL) Outcomes Reported Using the EORTC QLQ-C30 Questionnaire (questions 1 to 20)

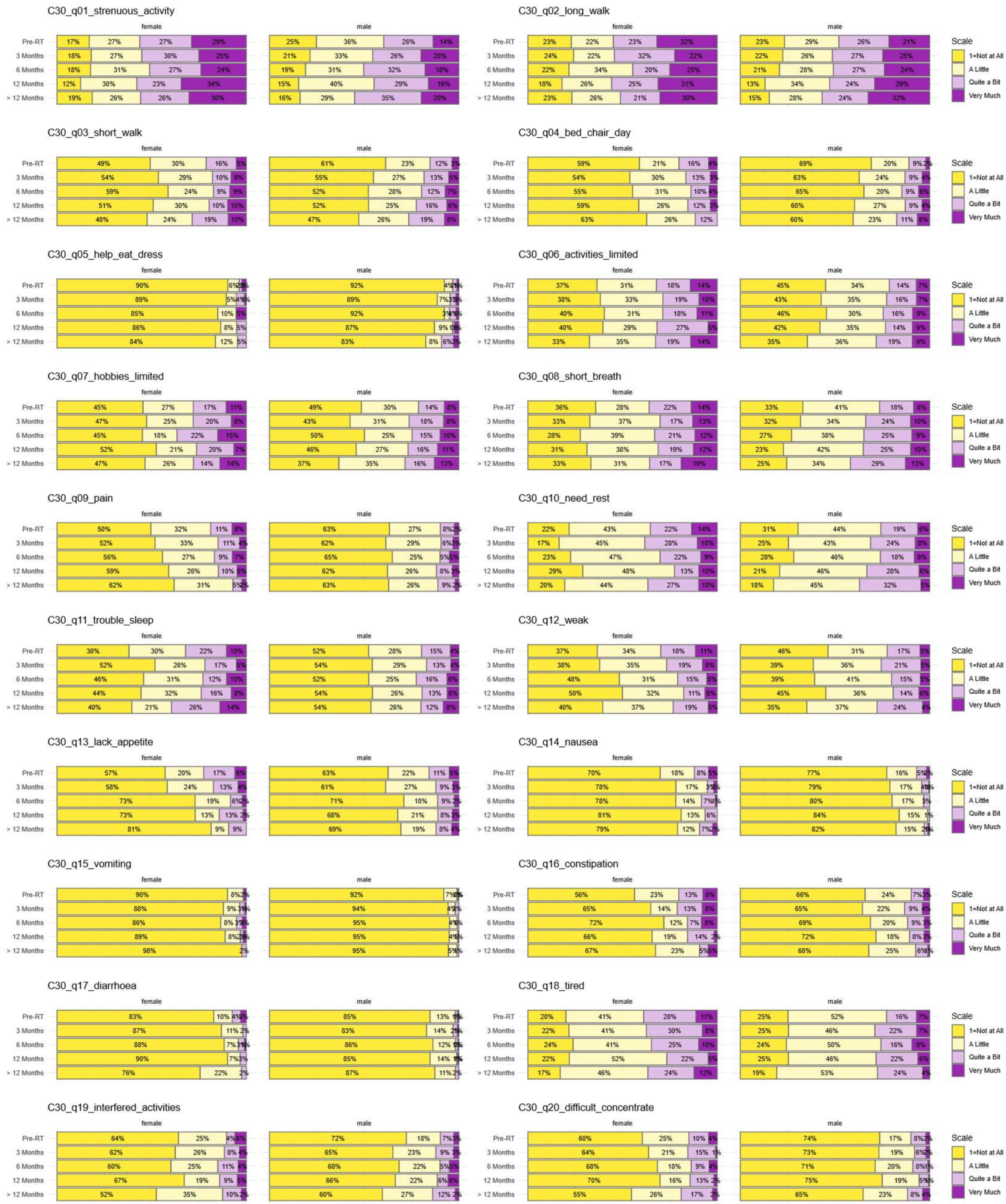

Supplementary Figure 2. Relative Frequencies of Health-Related Quality of Life (HRQoL) Outcomes Reported Using the EORTC QLQ-C30 Questionnaire (questions 21 to 30)

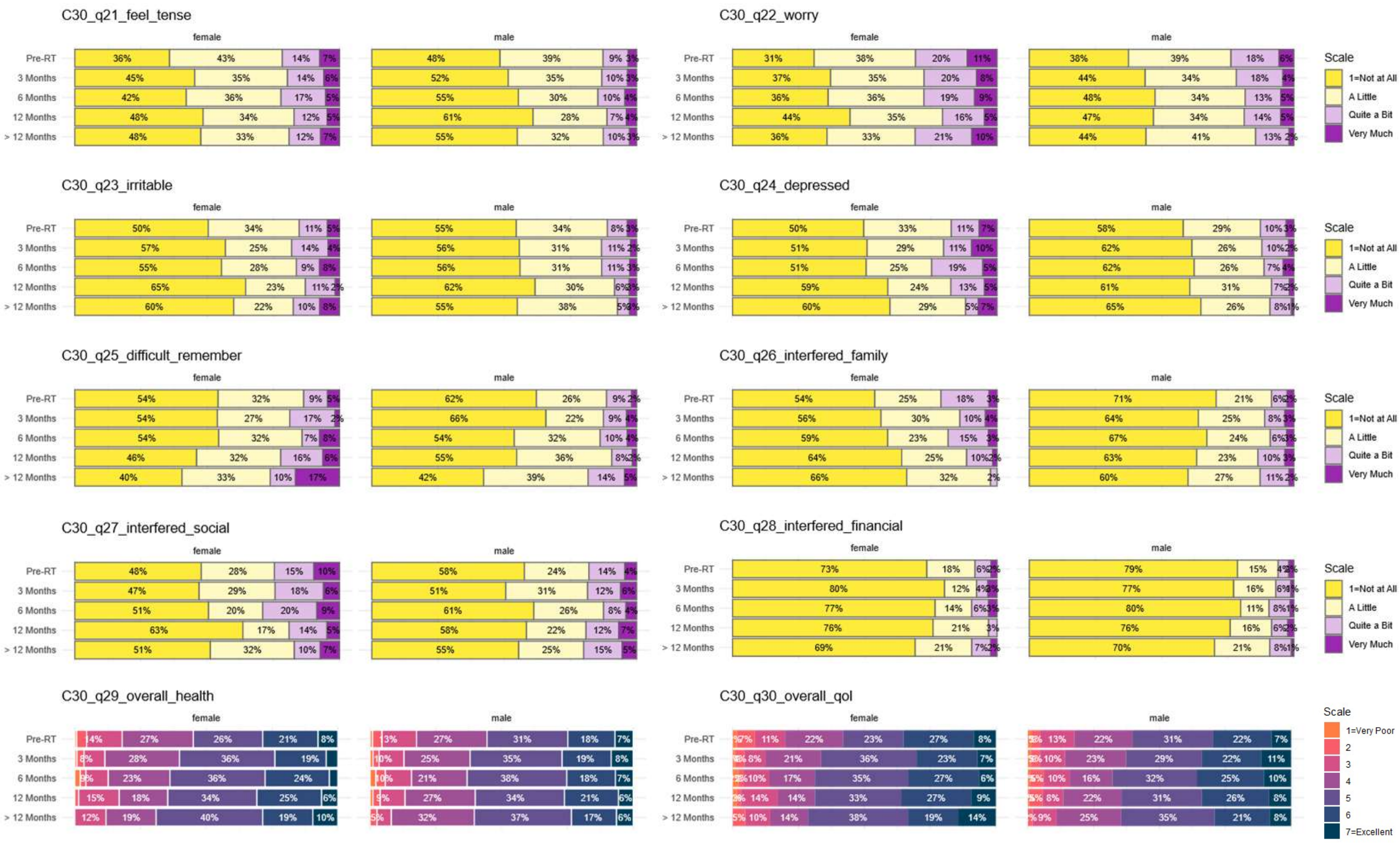

Supplementary Figure 3. Relative Frequencies of Patient-Reported Outcomes from the Multidimensional Fatigue Inventory (MFI)

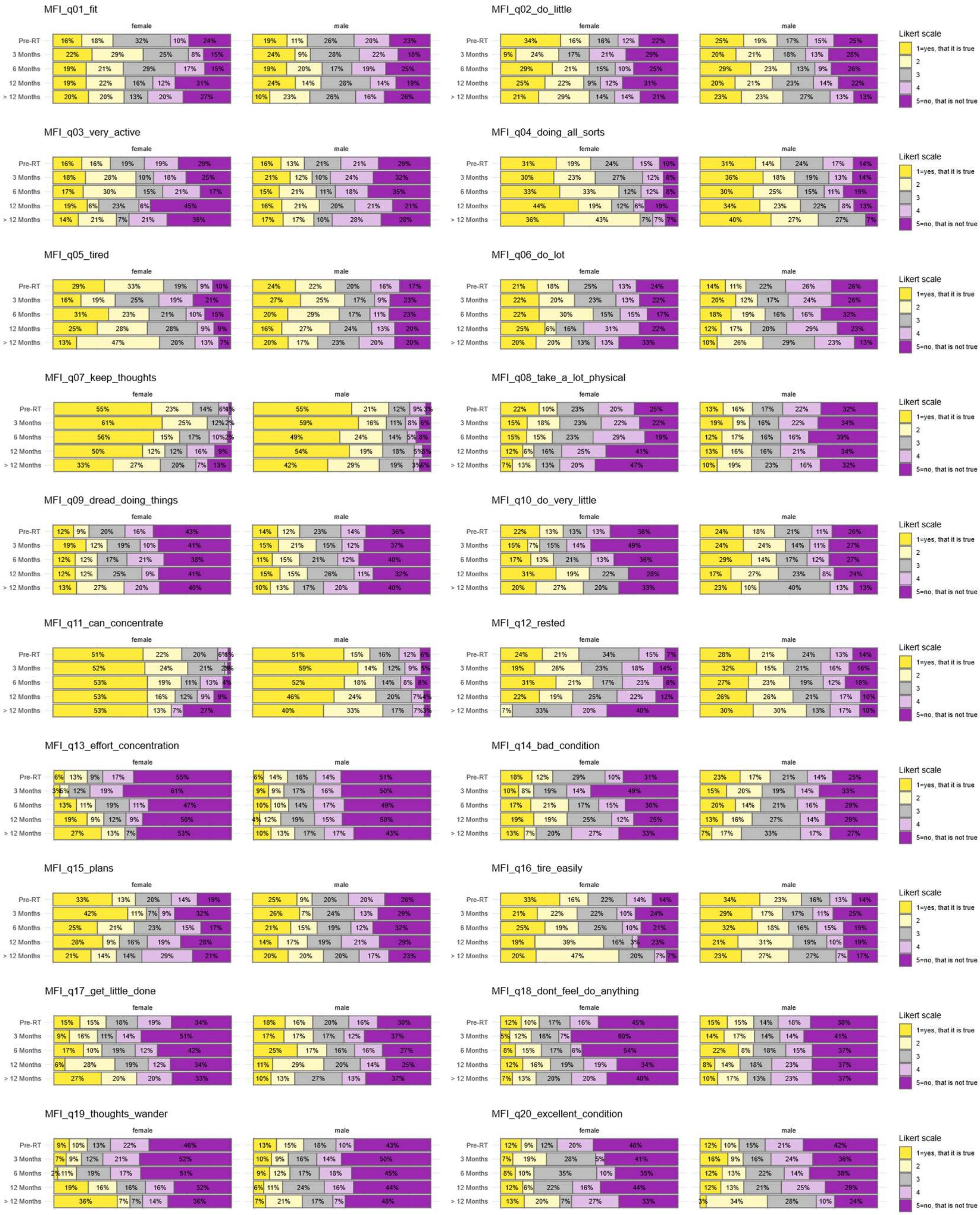

Supplementary Figure 4. Sex-Stratified Sankey Representation of CTCAE Grades of Adverse Events from Baseline to Late Follow-up in Lung Cancer Patients treated with Radiotherapy

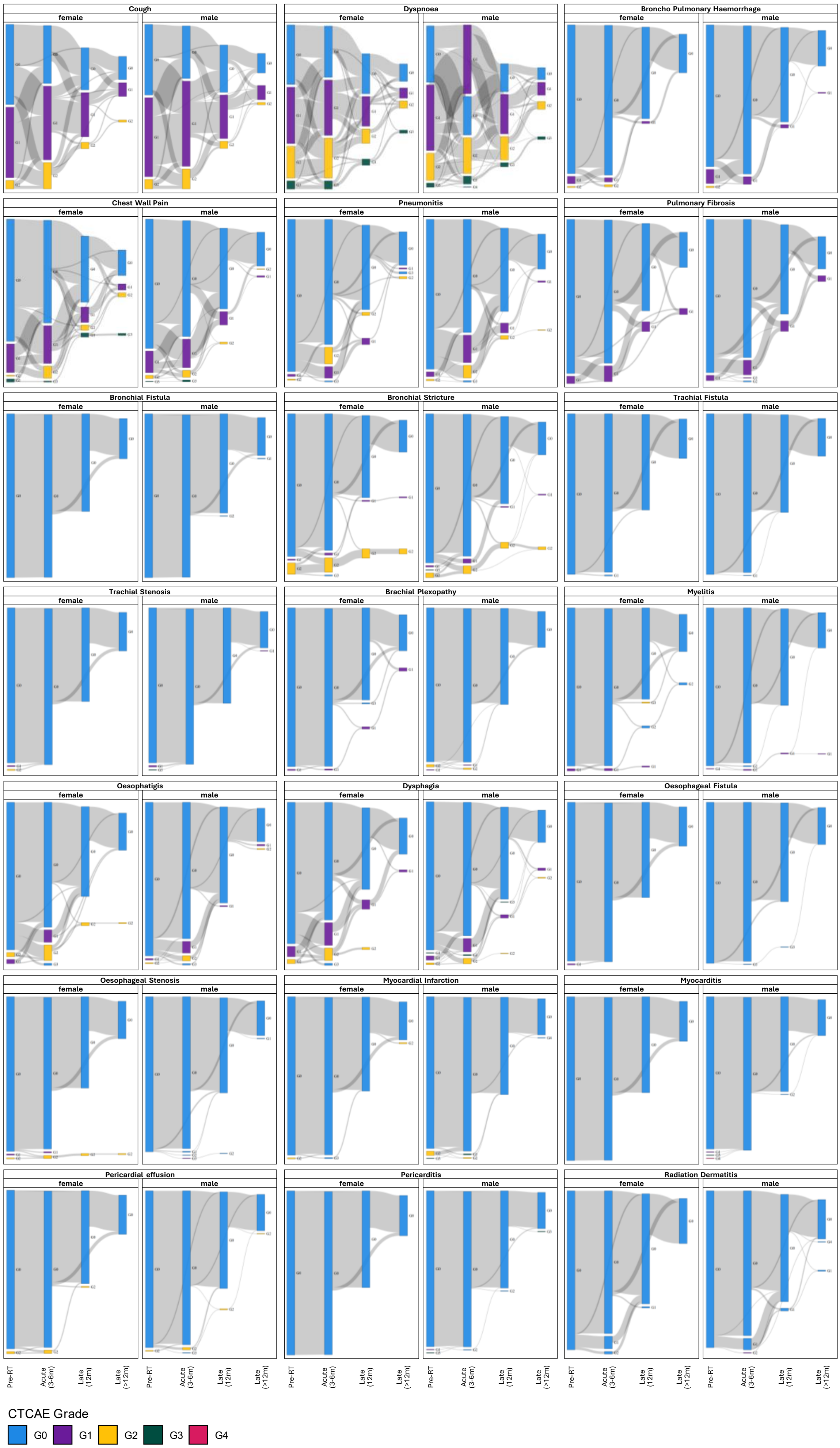

Supplement: Supplementary file 1 [file mmc1.pdf]
